# Nervonic acid and the long arc of therapeutic hope in X-linked adrenoleukodystrophy

**DOI:** 10.1016/j.neurot.2026.e00942

**Published:** 2026-06-27

**Authors:** Florian Eichler

**Affiliations:** MASSACHUSETTS GENERAL HOSPITAL, BOSTON, MA 02114, United States

**Keywords:** Lipid, Fatty acid, Peroxisome, Adrenoleukodystrophy, Adrenomyeloneuropathy

The history of X-linked Adrenoleukodystrophy (ALD) is, at its core, a story of both serendipity and persistence - of scientists following biochemical clues from the accidental discovery of very long chain fatty acids (VLCFA) to molecular precision, and of clinicians striving to translate those insights into meaningful therapies. The emergence of nervonic acid as a potential therapeutic strategy must be understood within this broader trajectory, one that has repeatedly oscillated between optimism and limitation.

The early observations by James Powers and colleagues in 1974, describing lipid inclusions in adrenal cells, marked the beginning of a lipid-centered understanding of ALD [1]. This was soon deepened by the work of Igarashi and Suzuki, who identified an excess of VLCFAs in cholesterol esters - an insight reportedly aided by an accidental overnight gas chromatography run [2]. Such moments of chance catalyzed a field that would come to define ALD by its biochemical hallmark: the accumulation of VLCFAs.

The work of Ann Moser and Hugo Moser later transformed VLCFAs from a biochemical curiosity into a clinically actionable biomarker [3]. Their demonstration that sustained reduction of VLCFAs could significantly lower the risk of the devastating childhood cerebral phenotype reframed ALD as a disease potentially modifiable through metabolic intervention [4]. This concept - dietary or biochemical modulation of lipid pathways - laid the intellectual groundwork for considering compounds like nervonic acid.

Yet, the path was not linear. Early hypotheses misidentified the underlying defect as VLCFA-CoA synthetase deficiency, only to be corrected with the discovery of the ABCD1 gene in 1993 [5]. This gene, encoding a peroxisomal transporter, clarified that ALD is fundamentally a disorder of impaired VLCFA degradation rather than synthesis. Animal models lacking this gene reproduced biochemical abnormalities but notably lacked the inflammatory demyelination seen in humans - highlighting a persistent gap between metabolic correction and clinical outcomes.

It is within this complex landscape that nervonic acid enters the conversation. In this issue of Neurotherapeutics, Kartha et al. demonstrate that nervonic acid can ameliorate the hallmark biochemical abnormalities of ALD in rodent plasma and brain [6]. As a monounsaturated very long chain fatty acid (C24:1), nervonic acid is a key component of myelin lipids. Its theoretical appeal lies in its dual potential: to modulate lipid composition and to support myelin integrity. Unlike earlier dietary approaches that focused primarily on reducing VLCFA levels, nervonic acid represents a more nuanced strategy - one that aims to rebalance lipid homeostasis rather than simply suppress a single biochemical marker.

However, history urges caution. The field has repeatedly demonstrated that lowering VLCFAs, while necessary, may not be sufficient [4]. The disconnect between biochemical correction and neurological stabilization remains one of the central challenges in ALD. Therapies targeting inflammation and oxidative stress - such as pioglitazone - have shown promise in preclinical models [7] but limited translation in human disease, particularly in adrenomyeloneuropathy (AMN) [8]. Similarly, advances in biomarkers, including C26:0 lysophosphatidylcholine, have improved prognostication without yet delivering definitive therapeutic breakthroughs. More recently, targeted inhibition of ELOVL1, the enzyme responsible for synthesis of VLCFA, has shown toxicity in preclinical models, presumably due to an excess reduction of VLCFA [9].

In this context, the value of nervonic acid must be evaluated in a dose dependent manner and not only as a standalone solution but also as part of a broader, combinatorial approach. Its role may be most impactful when integrated with strategies that address the multifactorial nature of ALD - genetic defect, metabolic dysfunction, neuroinflammation, and oxidative injury [10,11]. Moreover, the increasing precision of newborn screening and genetic diagnostics offers an opportunity to intervene earlier, when metabolic therapies are most likely to alter disease trajectory.

This history reminds us that progress in ALD has often come from reframing the problem rather than solving it outright. Nervonic acid, while not a panacea, represents such a reframing: a shift from reductionist thinking toward restoration of lipid balance and myelin health in the setting of evolving neurodegeneration and changing disease stages.

Ultimately, the question is not whether nervonic acid alone can treat ALD, but whether it can meaningfully contribute to a more nuanced and layered therapeutic strategy. If the past five decades have taught us anything, it is that incremental advances - grounded in deep biochemical understanding - are the true drivers of change. Nervonic acid deserves careful, rigorous evaluation within this tradition, carrying forward a legacy of inquiry that began with lipid inclusions under a microscope and continues today at the frontiers of metabolic and neurodegenerative disease.

## Disclosure

During the preparation of this work the author used ChatGPT to refine logical flow of commentary. After using this tool, the author reviewed and edited the content as needed and takes full responsibility for the content of the published article.

## Author contributions

The sole author (F.E.) is entirely responsible for the conceptualization, drafting, and critical revision of this commentary.

## Funding

This work was funded by NINDS/NCATS U54NS115052 and NINDS R61NS131928.

## Declaration of competing interest

The authors declare the following financial interests/personal relationships which may be considered as potential competing interests:

Reports a relationship with that includes:. Has patent pending to. If there are other authors, they declare that they have no known competing financial interests or personal relationships that could have appeared to influence the work reported in this paper.
